# Measuring serum oestrogen levels in breast cancer survivors using vaginal oestrogens: a systematic review

**DOI:** 10.1007/s10549-024-07364-0

**Published:** 2024-05-23

**Authors:** Antonia Pearson, Jill Chen, Haryana M. Dhillon, Belinda E. Kiely

**Affiliations:** 1 Sydney Medical School, University of Sydney, Camperdown, NSW Australia; 2 Psycho-Oncology Cooperative Research Group, Faculty of Science, School of Psychology, The University of Sydney, Camperdown, NSW Australia; 3 NHMRC Clinical Trials Centre, University of Sydney, Camperdown, NSW Australia

**Keywords:** Breast cancer, Genitourinary syndrome of menopause, Vaginal atrophy, Vaginal estrogen, Estrogen testing, Estradiol

## Abstract

**Purpose:**

Vaginal oestrogens can be used to treat genitourinary symptoms in women with early breast cancer. Studies evaluating vaginal oestrogens have commonly measured serum oestrogen levels as a surrogate marker of safety, but methods vary. We sought to summarise the data on serum oestrogen measurement in women with breast cancer using vaginal oestrogens to better understand the methods, levels and reliability.

**Methods:**

We searched Medline, Embase, CENTRAL, SCOPUS and CINAHL from inception to October 2023 for clinical studies where serum oestrogen was measured in women with a history of early breast cancer using vaginal oestrogens. Studies with a reported testing methodology were included.

**Results:**

Nine studies met the inclusion criteria for this systematic review. Methods used to measure oestradiol and oestriol in selected studies included mass spectrometry and immunoassays; several studies used more than one with variable concordance. Mass spectrometry detected oestradiol levels down to a lower limit between 1.0 pg/mL and 3.0 pg/mL. Immunoassays such as ELISA (enzyme-linked immunosorbent assay), ECLIA (enhanced chemiluminiscence immunoassay) and RIA (radioimmunoassay) had lower detection limits ranging between 0.8 pg/mL and 10 pg/mL. Studies were heterogeneous in testing techniques used, timing of testing, and the population including with subsequent varying results in the effect on oestrogens reported.

**Conclusions:**

Adopting consistent and standardised methods of measuring oestrogens in clinical trials involving women with early breast cancer on vaginal oestrogens is critical. Serum oestrogens are used as a surrogate marker of safety in this population, and good-quality data are necessary to enable clinicians and patients to feel confident in prescribing and taking vaginal oestrogens. Mass spectrometry, although more expensive, gives more reliable results when dealing with very low levels of oestrogens often found in women on aromatase inhibitors, compared to immunoassays.

## Introduction

Genitourinary Syndrome of Menopause (GSM) or vulvovaginal atrophy is reported in up to 75% of women after breast cancer (BC) [1]. Symptoms are more prevalent in women with BC compared to peers without [2, 3]. Chemotherapy, treatment-induced early and rapid onset of menopause and endocrine therapy can all contribute to GSM. Changes in genital tissues, with decreasing blood flow and secretions, increased pH, epithelial thinning and loss of elasticity occur from chronic oestrogen depletion [4]. GSM can negatively impact sexual function and health-related quality of life [5], presenting as follows: vaginal dryness; vaginal itch or burning; dysuria; urinary urgency; urinary incontinence; dyspareunia; loss of libido; dysfunction of arousal and orgasm; and increased urinary tract infections [4, 6].

Treatments for GSM include vaginal moisturisers and lubricants, oral or transdermal hormone replacement therapy, vaginal oestrogen therapies, and vaginal laser [7]. For women with a history of BC, particularly hormone receptor-positive (HR+) early breast cancer (EBC), systemic hormone-based therapies are not recommended due to concerns they increase BC recurrences by stimulating tumour growth [4]. However, vaginal oestrogens are considered safe for women with BC based on several studies showing no increased risk of de novo BC, [8–10] or recurrences [11–13]. International guidelines support use of vaginal oestrogens in women with BC, when non-hormonal interventions are unsuccessful in treating symptoms [14, 15]. Cold et al.’s cohort study [16] has renewed debate regarding vaginal oestrogen safety. Overall, there was no increased BC recurrences or mortality in women with EBC using vaginal oestrogens, but a subgroup analysis showed increased recurrences in women on aromatase inhibitors (AI) but not in women on tamoxifen [16], which may lead to some preferences for tamoxifen as a result.

To definitively establish safety, a large, prospective, randomised trial of vaginal oestrogens in women with BC assessing recurrences and mortality is required. Such a trial would require thousands of women and many years of follow-up, making it is unlikely to be conducted. Instead, measurement of serum oestrogen levels in women using vaginal oestrogens has been a key method of assessing safety.

The primary oestrogens are oestrone (E1), oestradiol (E2) and oestriol (E3). Available vaginal oestrogen preparations contain oestradiol and oestriol, so measurement of these oestrogens in the serum is of greatest interest. Oestradiol is the most frequently tested oestrogen. Oestriol is less potent and shorter acting than oestradiol and cannot be converted back into oestradiol [17, 18]. The problem with measuring oestrogen levels is the lack of clearly defined safe levels of serum oestrogen for women with BC. The normal range for median oestradiol in postmenopausal women ranges from 5 to 27 pg/ml [19, 20]. For women on AIs, which suppress oestradiol by up to 99% [20], serum oestradiol levels are typically lower with mean levels reported between 2 and 10 pg/ml [19, 20], and with ultrasensitive testing median oestradiol levels as low as < 0.1 pg/ml [20]. Previous studies used multiple methodologies for measuring oestrogens with varying sensitivity and reliability, making interpretation challenging. For example, results from pooled individual patient data from nine prospective case–control studies revealed an increased relative risk of BC recurrence of 1.29 (95% CI, 1.15–1.44, *p* < 0.001) for every doubling of oestradiol (E2) concentration [21]. One study defined a prolonged rise of oestradiol as > 10 pg/mL and > 10 pg/mL above baseline on 2 consecutive blood tests > 2 weeks apart [22].

Any method used to measure serum oestrogens in clinical trials and practice must meet these criteria: ability to detect very low levels; high specificity; and reproducibility [23]. Commercially available oestradiol testing is often unable to detect very low levels of oestradiol (E2) induced by AI. Mass spectrometry (liquid chromatography mass spectrometry [LC-MS] or gas chromatography mass spectrometry [GC-MS]) and immunoassays have been used in clinical trials, but do not always show concordance [24]. Mass spectrometry appears more accurate at detecting low levels of oestrogens [20, 23, 25]. However, access and cost prohibit use outside clinical trials.

Our systematic review aimed to (i) identify methods used to test oestradiol, oestriol and other hormones; (ii) summarise E2 and E3 levels reported; (iii) assess the quality of reported assays, in women with HR+ EBC using vaginal oestrogens.

## Methods

Our systematic review was conducted according to PRISMA guidelines [26]. Searches were conducted in October 2023 using five databases: Medline, Embase, CENTRAL (Cochrane Register of Controlled Trials), SCOPUS, and CINAHL (Cumulative Index to Nursing and Allied Health Literature). Table 3 shows search terms used. Reference lists of relevant studies were hand searched and a Google Scholar search performed to identify additional studies.

Eligible studies included randomised controlled trials (RCTs) and other clinical intervention studies. Study inclusion criteria were postmenopausal women with a history of HR+ EBC receiving any form of vaginal oestrogen treatment. To be included, oestradiol and/or oestriol had to be measured and techniques and testing parameters reported. Studies involving systemic (oral or transdermal) oestrogen treatments were excluded. Conference abstracts were permitted where required information was accessible.

Following database searches, studies were imported to Covidence [27], and duplicates identified. Two authors (AP; JC) independently screened titles and abstracts to determine eligibility. Full manuscripts were independently reviewed by both authors, with disagreements reviewed by BK.

Data extracted included design, duration, inclusion criteria, menopausal status and age, use and type of endocrine therapy, type of vaginal oestrogen (including dose, frequency, duration), control group, sample size, oestrogen testing timepoints, oestrogens measured (for each: level, testing method, unit of measurement, detection limits, normal range), other sex hormones measured. Study quality and risk of bias were to be assessed if possible.

## Results

**Fig. 1:**
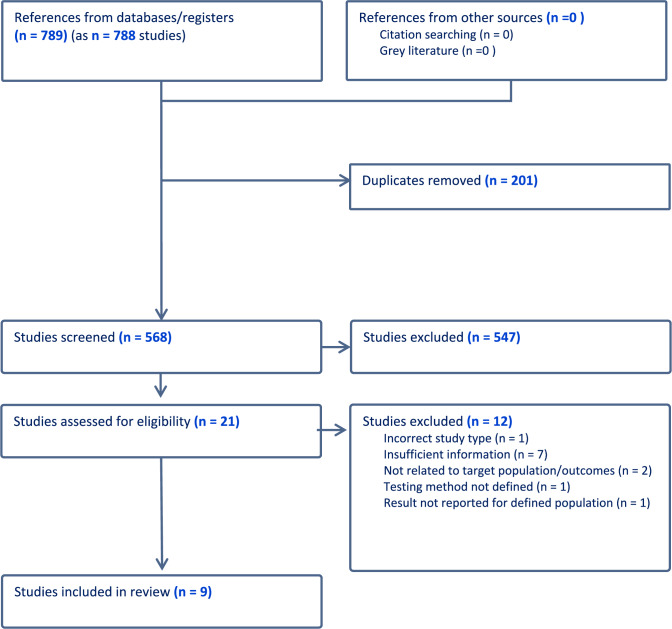
PRISMA flow diagram

The PRISMA flow diagram (Fig. 1) depicts 789 citations identified, of which 201 were duplicates and 547 excluded on title and abstract screening. After full-text review, nine studies [22, 28–36] met the inclusion criteria (See Table 1). Of these, three were RCTs, two case–control studies, and four single-arm studies. Oestrogen testing methodology is outlined in Table 2.

**Table 1:** Summary of study characteristics

Study	Study type	N	Population	Intervention or controls	Intervention frequency and duration	Measurement timepoints	Effect of VE on oestrogens (E2 and E3)	Major limitations
Biglia et al. [28]	Prospective case–control study	26	Hormone receptor positive EBC on tamoxifen ± ovarian suppression	Group A: oestriol cream (0.25 mg) Group B: oestradiol pessary 12.5mcg Group C: polycarbil based moisturiser	Group A: twice per week for 12 weeks Group B: twice per week for 12 weeks Group C: twice per week for 12 weeks	Baseline and 12 weeks	No change in mean E2 or E3	● Small sample size ● Not randomised ● Women on AIs excluded from intervention group ● Test for oestrogens not as sensitive as other studies
Donders et al. [29]	Prospective single-arm study	16	Hormone receptor positive EBC on AI > 6 months	100 million acidophilus KS400 and oestriol 0.03 mg combination tablet	Daily for 4 weeks then 3 times per week for 8 weeks	0.5 h before and 0.5, 1, 2, 3, 4, 6, 8, and 24 h after tablet insertion and at weeks 2, 4, 8 and 12	Rise in E3 at 2 weeks, decreasing by 4 weeks	● Very small sample size ● Single arm study
Kendall et al. [32]	Prospective single-arm study	6	Hormone receptor positive EBC on AI*	Oestradiol pessary 25mcg	Daily for 2 weeks then 2 times per week for 10 weeks	Baseline, and weeks 2, 4, 7–10, and 12	Rise in E2 at 2 weeks, decreasing by 4 weeks Persistent rise in E2 in 2 patients at 7–10 weeks	● Very small sample size ● Single arm study ● Heterogeneity in testing schedules ● Variable reported oestrogen levels
Melisko et al. [22]	Prospective randomised study	76	Hormone receptor positive EBC on AI > 30 days	Group A: Intravaginal testosterone cream 1% (0.5 g) (*N = *36) Group B: vaginal ring (7.5mcg oestradiol released every 24 h) (*N = *40)	Group A: Daily for 2 weeks then 3 times per week for 10 weeks Ground B: every 90 days	Baseline, weeks 4 and 12	37% had elevated E2 at baseline 4 participants had rise in E2 at 4 weeks No patients had a persistent rise	● Some variability in results between testing methods ● Significant discordance rare
Niravath et al. [33]	RCT (sub-study from REVIVE RCT)	8	Hormone receptor positive EBC on AI	Group A: vaginal ring (7.5mcg oestradiol released every 24 h) Group B: polycarbil based moisturiser	Group A: every 90 days for 6 months Group B: 3 times per week for 6 months	Baseline, weeks 2, 4, 8,12,16 and 20	Not assessable	● Very small sample size ● Heterogeneity of testing methods ● Discordant results
Pfeiler et al. [34]	Prospective single-arm study	10	Hormone receptor positive EBC on AI > 1 year	Oestriol 0.5 mg pessary	Daily for 2 weeks	Baseline, 15 days	No rise in E2 or E3	● Very small sample size ● Single arm study ● Short study duration ● ECLIA poor sensitivity ● GC–MS detection limit unclear
Sanchez-Rovira et al. [35]	RCT	61	Hormone receptor positive EBC on AI > 6 months	Group A: 0.005% oestriol gel (0.5 mg oestriol) (*N = *50) Group B: placebo (moisturising gel) (*N = *11)	Daily for 3 weeks then 2 times per week for 11 weeks	Baseline, and week 1, 3, 8 and 12	Rise in E3 after 1 week, normalising by week 12 No change in E1 or E2	None
Streff et al. [36]	Prospective and retrospective single-arm study	14	Hormone receptor positive EBC on AI	Vaginal ring (7.5mcg oestradiol released every 24 h)	Every 90 days for 16 weeks	Baseline, week 4 and 16	Rise in E2 at 4 weeks No rise in E2 at 16 weeks	● Very small sample size ● Single arm study ● Heterogenous testing methods ● Detection limits unclear
Wills et al. [30]	Prospective case–control study	48	Hormone receptor positive EBC or high risk for BC on endocrine therapy and Cases: had to be on vaginal oestrogens > 3 months Controls: had to be on AI	Group A: pessary (oestradiol 25mcg) (*N = *14) Group B: vaginal ring (7.5mcg oestradiol released every 24 h) (*N = *10) Group C: no intervention (*N = *24)	Group A: 2 times per week for 60 days Group B: every 90 days	Group A: pre-insertion, 12 h post insertion Group B: pre-insertion, 30 days, 60 days post insertion Group C: once	Pre-insertion: pessary: E2 not elevated Ring: 37% E2 level elevated compared to controls After insertion E2 levels rose in both groups Persistent elevation in Ring group at 60 days compared to controls	● Small sample size for each intervention ● Heterogeneous population ● No randomisation

*RCT* randomised controlled trial, *EBC* early breast cancer, *BC* breast cancer, *AI* aromatase inhibitor, *VE* vaginal oestrogen

*This study also included an additional patient with metastatic breast cancer who used premarin cream (conjugated oestrogens)

**Table 2:** Summary of oestrogen testing methods

Study	Oestradiol (E2) testing method	Oestradiol (E2) detection limits	Oestriol (E3) testing method	Oestriol (E3) detection limits	Other hormones tested
Biglia et al. [28]	RIA	5.0 pg/mL	Not tested		E1, FSH, LH, Testosterone, SHBG
Donders et al. [29]	Highly sensitive GC–MS	1.0 pg/mL	Highly sensitive GC-MS	10.0 pg/mL	E1, FSH, LH, SHBG
Kendall et al. [32]	RIA	3.0 pmol/L (0.82 pg/mL)	Not tested		FSH, LH
Melisko et al. [22]	RIA and ultrasensitive LC–MS	RIA: 3.0 pmol/L (0.82 pg/mL) LC-MS sensitivity not defined	Not tested		Baseline FSH, testosterone (for those on testosterone arm)
Niravath et al. [33]	ELISA and Small number additionally used LC–MS/MS	ELISA: 3.0 pg/mL LC–MS/MS: 1.0 pg/mL	Not tested		Not tested
Pfeiler et al. [34]	ECLIA and GC–MS	ECLIA: 10.0 pg/mL GC–MS detection limit unclear	ECLIA and GC–MS	Unclear	FSH, LH
Sanchez-Rovira et al. [35]	Ultra-sensitive LC-MS/MS	3.0 pg/mL	Ultra-sensitive LC-MS/MS	1.0 pg/mL	E1, FSH, LH
Streff et al. [36]	Tandem mass spectrometry for prospective, for retrospective: mixture of mass spectrometry or RIA or ECLIA	Unclear	Not tested		Not tested
Wills et al. [30]	RIA	3.0 pmol/L (0.82 pg/mL)	Not tested		Not tested

*E1* Oestrone, *ECLIA* Electro-chemoluminescence immunoassay, *ELISA* Enzyme-linked immunosorbent assay, *FSH* Follicle stimulating hormone, *GC*-*MS *Gas Chromatography Mass Spectrometry, *LC*-*MS* Liquid Chromatography Mass Spectrometry, *LH* Lutenising hormone, *RIA* Radioimmunoassay and *SBHG* Sex binding-hormone globulin

Methods used to measure oestradiol and oestriol in selected studies included mass spectrometry and immunoassays; several studies used more than one with variable concordance [22, 33]. Mass spectrometry detected oestradiol levels down to a lower limit between 1.0 pg/mL and 3.0 pg/mL [22, 29, 33–36]. Immunoassays such as ELISA (enzyme-linked immunosorbent assay), ECLIA (enhanced chemiluminiscence immunoassay) and RIA (radioimmunoassay) had lower detection limits ranging between 0.8 pg/mL and 10 pg/mL [22, 28, 30, 32–34, 36].

The highest quality and largest study was Sanchez-Rovira et al.’s trial of vaginal oestriol [35]. Sixty-one women with HR+ EBC taking an AI for > 6 months were randomised to a 0.005% oestriol gel (50mcg oestriol per application) or placebo moisturising gel daily for 3 weeks then twice weekly for 9 weeks. Serum oestrogen levels were measured at baseline, 3, 8, and 12 weeks via ultrasensitive LC-MS (detection limit of 3 pg/mL for oestradiol and 1 pg/mL for oestriol) [37]. There was no significant rise in E2 detected. A small rise in E3 was detected in the treatment group in the first 3 weeks returning to same level as the placebo group by 12 weeks (median, [interquartile range IQR], E3 = 0.5 pg/mL [0.5 to 7.3] in the treatment group and 0.5 pg/mL [0.5 to 0.5] placebo group, *p* = 0.140). Oestrone, follicle stimulating hormone (FSH) and luteinising hormone (LH) had no significant changes detected at any timepoint.

The REVIVE study [33], an ongoing open-label RCT, reported serum hormone levels for the first eight participants. This study compares a vaginal oestradiol ring (Estring®) which releases approximately 7.5mcg oestradiol daily for 90 days to a polycarbophil-based vaginal moisturiser (Replens ™) in women with HR+ EBC taking an AI. Two different ELISA kits were used to quantify oestradiol (E2) with a detection limit of 3 pg/mL. There was concordance between two ELISA kit measurements for 5/6 patients, but one had different results: E2 < 3 pg/mL versus E2 67 pg/mL. Samples from three patients were analysed using LC-MS/MS with a detection limit of 1 pg/mL. The values of E2 between different testing methods (ELISA and LC-MS/MS) varied markedly. One patient had higher readings of E2 on both ELISA kits compared to LC-MS/MS, and the degree of elevation varied between the two kits. Another patient had elevation in E2 at 2 weeks on LC-MS/MS, not detected on ELISA. The third patient was taking exemestane, an AI known to cause aberrant results via immunoassays due to cross-reactivity [20, 38]. Her results showed the anticipated rise in E2 on ELISA, but corresponding LC-MS/MS testing showed an elevated level of E2 at baseline but levels < 2 pg/mL at all other timepoints, attributed to a laboratory error. Although the RCT design is sound, this sub-study is fraught with issues: small sample size (*N* = 8), heterogeneity of testing methodologies, and discordant results.

Melisko et al. [22] conducted a randomised open-label phase 2 study comparing vaginal testosterone cream to a vaginal oestradiol ring (Estring®) in 76 women with HR + EBC on AI for > 30 days. The vaginal testosterone cream was used daily for 2 weeks then three times per week for 10 weeks for the first 24 participants then changed to three times per week for the 12-week study duration for the subsequent 12 participants. Both RIA and ultrasensitive LC-MS methods were used to measure oestradiol (E2) with a detection limit of 3 pmol/L (0.82 pg/mL) for RIA and no reported detection limit for LC-MS. The authors predefined < 10 pg/mL as the expected postmenopausal level of oestradiol, and the primary safety endpoint was defined as < 25% of participants having a persistent elevation of E2 (> 10 pg/mL and at least > 10 pg/mL above baseline on two consecutive blood tests more than two weeks apart) and was met for both arms in this study. 63 participants had E2 measured with both methods and surprisingly of these, 25 (40%) had baseline E2 above the 10 pg/mL threshold with LC-MS and 9 (14%) with RIA. In the oestradiol ring arm, 4/35 participants had a transient rise in E2 (range 11–29 pg/mL) but none had persistent elevation. No differences were detected in mean E2 levels between LC-MS and RIA at baseline (17.7 [SD 28.5] pg/mL and 17.9 [SD 44.1] pg/mL, respectively) or at week 4 (7.8 [SD 15.0] pg/mL and 2.9 [SD 13.4] pg/mL, respectively). There was some variability of reported E2 levels between RIA and LC-MS on review of matched samples, but discordant E2 elevation was rare (2/63 participants with matched samples). Similar to REVIVE [33], Melisko et al. found that LC-MS was more likely than RIA to detect an early rise in E2, 19% of participants had elevated E2 at 4 weeks with LC–MS vs. 1.5% with RIA. This study had a reasonable sample size, with most patients (63/76) having matched samples, allowing greater reliability of concordance assessment for testing methods. Although very discordant results were rare, there was still variability and RIA failed to detect early rises in E2 which were detected with LC-MS.

A prospective, open-label, single-arm study by Kendall et al. which showed a persistent rise in oestradiol (E2) levels, [32] is often used as a basis for concern regarding safety of vaginal oestrogens. In this study, six women with HR+ EBC on AI for > 6 months used a 25mcg vaginal oestradiol pessary (Vagifem®) daily for two weeks, then twice weekly for 10 weeks. Oestradiol (E2) was measured using RIA with a detection limit of 3 pmol/L (0.82 pg/mL). One participant on exemestane had high baseline levels of E2 attributed to cross-reactivity with immunoassays [38]. E2 levels increased at two weeks in 5/6 participants (from median 0.82 pg/mL at baseline to 19.61 pg/mL) and decreasing to median 4.36 pg/mL at 4 weeks and stayed at this level for most participants. However, two participants had persistently raised E2 when tested between 7 and 10 weeks (37.32 pg/mL and 59.65 pg/mL), one of whom was on exemestane. This study has several limitations including a very small sample size, no randomisation, heterogeneity in testing schedules, and variable reported oestrogen levels. The study also used a 25mcg oestradiol pessary, which has been subsequently replaced by a lower dose 10mcg preparation (Vagifem Low®).

Donders et al. [29] enrolled 16 women with HR+ EBC on AI for > 6 months to take a vaginal tablet containing 100 million acidophilus KS400 and 0.03 mg oestriol (Gynoflor®) daily for 4 weeks, then three times per week for 8 weeks. The authors used a highly sensitive GC-MS to quantify oestradiol (E2) and oestriol (E3) with a detection limit of 1 pg/mL and 10 pg/mL, respectively. As expected, there was no rise in E2 (as oestriol does not back convert to oestradiol [17, 18]), but there was a small transient rise in E3, most pronounced after the first dose. Oestrone, LH, FSH and sex hormone-binding globulin (SBHG) were tested, with no change detected except in FSH which had a small but significant decrease at 4 weeks. Again, this study is limited by a very small sample size and its single-arm design.

A short study by Pfeiler et al. [34] assessed use of oestriol 0.5 mg pessaries (Ovestin®) daily for two weeks in 10 women with HR + EBC on anastrozole. Oestradiol (E2) was quantified at baseline and 2 weeks using both ECLIA (detection limit of 10 pg/mL) and GC-MS (unreported detection limit). No significant change was reported for E2 or E3, but mean FSH and LH levels decreased significantly from baseline to 2 weeks (LH − 10.8%, *p* = 0.02; FSH − 12.8%, *p* = 0.01). This is a very small, single-arm study of two-week duration; thus, the duration of effect on FSH and LH remains unknown. Both ECLIA and GC-MS were performed, but detection limits were much higher for ECLIA than in other studies, so small rises in E2 could have been missed.

Biglia et al.’s [28] non-randomised three-arm study compared 0.25 mg oestriol cream, 12.5mcg oestradiol pessaries (Vagifem®), and a vaginal moisturiser (Replens™) in 26 women with HR + EBC on endocrine treatment. Unlike other studies reviewed, women on AI were not permitted in the vaginal oestrogen groups (but permitted in moisturiser group). RIA was used to quantify oestradiol (E2) with a detection limit of 5 pg/mL. No significant difference was found in E2 levels from baseline to week 12 or additionally, in E3, E1, LH, FSH, testosterone and SHBG. Although there were no reported issues with E2 RIA testing, the lower detection limit of 5 pg/mL is not as sensitive as methods used in other studies. Given most patients were not on an AI, the impact of small rises in oestradiol is less concerning.

Wills et al.’s prospective case–control study enrolled 48 women with HR+ EBC or an increased risk of developing BC (all on endocrine therapy) and compared cases of those on vaginal oestrogens for > 3 months (25mcg oestradiol pessary twice weekly or vaginal oestradiol ring inserted every 90 days), with a no vaginal oestrogen control group [30]. Oestradiol (E2) levels were measured using RIA with a detection limit of 3 pmol/L (0.82 pg/mL). For women using oestrogen pessaries for > 3 months, pre-insertion levels of E2 were not elevated compared to controls, although 12 h post-insertion E2 levels were raised. For those using the oestrogen ring for > 3 months, pre-insertion mean E2 levels were already elevated compared to controls suggesting a persistent elevation in E2 in this group. This study implemented a novel approach testing oestrogen levels pre- and post-insertion in women who had used vaginal oestrogens for > 3 months, with the intent of capturing whether persistent elevation in oestrogens occurred before insertion and after.

Streff et al. conducted a prospective study of vaginal oestradiol rings (Estring®) in women with HR+ EBC on AI [36]. This study included 8 prospective participants and 6 retrospective participants who had oestradiol (E2) quantification via tandem mass spectrometry or ECLIA with a variety of laboratories and reference ranges. Baseline E2 levels were in the expected range, but after commencing the oestradiol ring, 6/8 prospective participants had a transient rise in E2 (at week 4) which returned to baseline levels by week 16. The study quality was low due to small sample size, heterogeneous testing methods and no clear definition of the detection limits used.

Due to the heterogeneity of studies included in this systematic review, a formal bias assessment was not possible.

## Discussion

We identified a variety of testing methodologies used to quantify serum oestradiol (E2) and oestriol (E3) from included studies. Some used multiple techniques with mixed concordance across testing methods. Overall, the quality of studies was poor with considerable heterogeneity of testing techniques, timing and populations. There was also a mixture of concordant and discordant results when more than one methodology was used. This creates a confusing landscape to determine the impact vaginal oestrogens may have on oestrogen levels in women with BC. In addition, the impact of small, and often transient elevations in serum oestrogen levels on outcomes of women with BC remains unknown.

Folkerd et al. [39] cautioned against false interpretations from studies testing serum oestrogens with potentially erroneous quantification methods. The REVIVE study highlights challenges of using different testing methodologies for measurement of low levels of oestradiol (E2), with discordant oestrogen levels between methods [33].

Immunoassay methodologies (RIA, ECLIA, ELISA) can detect low levels of oestrogens, but are prone to inaccuracies [39], and cannot detect levels as low as mass spectrometry. Caution is required for patients taking exemestane, an AI known to falsely elevate oestradiol levels when immunoassays are used [38]. Immunoassays appear less likely to detect early rises in oestradiol levels in women using vaginal oestrogens compared to mass spectrometry [22, 33]. A recent study of women with EBC on letrozole showed no concordance between oestrogen levels measured with immunoassays compared to mass spectrometry [40]. Given multiple confounding issues with results from sensitive immunoassays, mass spectrometry appears the most reliable testing methodology in women with BC on vaginal oestrogens, particularly those on AI.

Vaginal oestrogen formulations varied considerably between included studies. Kendall et al. [32] and Wills et al. [30] used 25mcg oestradiol tablets (Vagifem®), replaced by lower dosage 10mcg formulation (Vagifem® Low), which remains effective for symptom relief with less systemic absorption [41, 42]. Oestradiol is also the oestrogen used in the ring (Estring®), whereas the other formulations were oestriol based, a less potent oestrogen which cannot be converted to oestradiol [17, 18].

Oestrogen levels increase after initiation of vaginal oestrogens due to a denuded vaginal epithelium, tapering over time as the epithelium recovers and absorption decreases [43, 44]. This transient rise was demonstrated in several studies [22, 29, 30, 32, 35, 36]. When considering the impact of vaginal oestrogens on BC recurrence risk, transient raised oestrogen levels are less concerning than a persistent rise. Oestrogen levels should be measured after vaginal oestrogens have been used for a few months. Of studies showing a persistent rise in oestrogen [30, 32], both assessed women using higher dose vaginal oestradiol products than typically recommended in women with BC. Wills et al. [30] showed persistent elevations of oestradiol (E2) at 60 days post-insertion in women using an oestradiol ring; however, in the oestradiol 25mcg tablet group, there were no elevations in oestradiol after 3 months of treatment, suggesting that any rise in E2 post-oestradiol tablet insertion is not persistent. Contrastingly, Kendall et al. [32] found persistently elevated E2 in two patients using 25mcg vaginal oestradiol tablets for 7–10 weeks, but no week 12 test was performed. This may reflect a true significant rise in E2 but may be due to inaccuracies of RIA.

Several studies in our systematic review are of low quality, and reasons include small sample size; retrospective design; mixed methodologies for testing oestrogens; inconsistencies in timing of oestrogen testing; lack of randomisation; and frequent lack of a control group. Despite significant limitations, these studies are often used to justify withholding vaginal oestrogens from women experiencing GSM because concerns use could increase BC recurrence risk.

It is essential that larger, more robust, and reliable studies are performed for this population. This includes a more uniform approach to oestrogen testing in clinical trials and practice. A better understanding of oestrogen levels and fluctuations over time in postmenopausal women with EBC not using vaginal oestrogens is essential; future studies should include a control group. Larger RCTs are needed to demonstrate the impact of longer-term use of vaginal oestrogens on oestrogen levels, given factors potentially influencing them including adherence, testing methodology, sample storage, timing of testing, and cross-reactivity with immunoassays. Relying on small studies to suggest a safety signal is problematic.

Limitations of our systematic review include the small number of included studies and heterogeneity of their design, size, intervention, and testing methods. Consequently, we were unable to perform a formal quality and bias assessment.

## Conclusion

Inconsistent measurement and reporting of serum E2 and E3 levels in women with EBC on endocrine therapy creates uncertainty in safety of vaginal oestrogens, generating reluctance in clinicians to prescribe and patients to use vaginal oestrogens, even when symptoms are severe. More robust and standardised methods of measuring E2/E3 are critical to improve our understanding of the impact of vaginal oestrogens on serum oestrogen levels. In the absence of prospective randomised trials assessing BC outcomes, measurement of serum oestrogens remains the main method of assessing safety. Good quality data are required to increase confidence of women and clinicians worldwide.

## Data Availability

No datasets were generated or analysed during the current study.

## Appendix

See Table

**Table 3:** Search terms

Database	Search	Results
MEDLINE 1946 to October 2023	exp Breast Neoplasms/ OR (Breast* adj2 (cancer* or tumo?r* or neoplas* or malignan* or carcinoma* or adenocarcinoma* or choricarcinoma* or metastat* or sarcoma* or teratoma or onlocog*)).mp AND Estrogens/ OR (estrogen* or oestrogen*).mp OR Estradiol/ OR Estriol/ OR (oestradiol or estradiol or oestriol or estriol).mp OR (E2 or E3).tw AND (topical* or vaginal* or gel* or cream* or pessary*).mp OR administration, topical/ or administration, intravaginal/ OR Gels OR Pessaries/ AND Postmenopause/ OR Post?menopause*.mp OR ovarian suppression*.mp	186
EMBASE 1947 to October 2023	exp breast cancer/ OR Breast* adj2 (cancer* or tumo?r* or neoplas* or malignan* or carcinoma* or adenocarcinoma* or choricarcinoma* or metastat* or sarcoma* or teratoma or onlocog*)).mp AND estrogen/ OR (oestrogen or oestrogen).mp OR estradiol/ OR estriol/ OR (oestradiol or estradiol or oestriol or estriol).mp OR (E2 or E3).tw AND (topical* or vaginal* or intravaginal* or gel* or cream* or pessary*).mp OR topical treatment/ OR gel/ OR vagina pessary/ AND postmenopause/ OR Post?menopause*.mp OR ovarian suppression*.mp AND limit to human and female	376
Cochrane Central Register of Controlled Trials October 2023	exp Breast Neoplasms/ OR (Breast* adj2 (cancer* or tumo?r* or neoplas* or malignan* or carcinoma* or adenocarcinoma* or choricarcinoma* or metastat* or sarcoma* or teratoma or onlocog*)).mp AND Estrogens/ OR (estrogen* or oestrogen*).mp OR Estradiol/ OR Estriol/ OR (oestradiol or estradiol or oestriol or estriol).mp OR (E2 or E3).tw AND (topical* or vaginal* or gel* or cream* or pessary*).mp OR administration, topical/ or administration, intravaginal/ OR Gels/ OR Pessaries/ AND Postmenopause/ OR Post?menopause*.mp OR "ovarian suppression*".mp	101
SCOPUS October 2023	(TITLE-ABS-KEY ( ( breast* W/2 ( cancer* OR tumo?r* OR neoplas* OR malignan* OR carcinoma* OR adenocarcinoma* OR choricarcinoma* OR metastat* OR sarcoma* OR teratoma OR onlocog*))) AND TITLE-ABS-KEY ( estrogen* OR oestrogen* OR oestradiol OR estradiol OR oestriol OR estriol OR e2 OR e3) AND TITLE-ABS-KEY ( topical* OR vaginal* OR gel* OR cream* OR pessary*) AND TITLE-ABS-KEY ( post?menopause* OR "ovarian suppression*"))	35
CINAHL October 2023	(MH "Breast Neoplasms+ ") OR (Breast* N2 (cancer* or tumo?r* or neoplas* or malignan* or carcinoma* or adenocarcinoma* or choricarcinoma* or metastat* or sarcoma* or teratoma or onlocog*)) AND (MH "Estrogens+ ") OR estrogen* or oestrogen OR (MH "Estradiol+ ") OR (MH "Estriol") OR oestradiol or estradiol or oestriol or estriol OR e2 or e3 AND topical* or vaginal* or gel* or cream* or pessary* OR (MH "Administration, Topical+ ") OR (MH "Administration, Intravaginal") OR (MH "Gels") OR (MH "Pessaries") AND (MH "Postmenopause") OR Post?menopause* OR "ovarian suppression*"	91

Exp or+ denotes explosive search,.mp denotes search as keyword, / denotes search as MeSH descriptor,.tw denotes search in title and abstract only, MH denotes a search for subject heading

3

## Abbreviations

AI: Aromatase inhibitor
BC: Breast Cancer
E1: Oestrone
E2: Oestradiol
E3: Oestriol
EBC: Early breast cancer
ECLIA: Enhanced chemiluminiscence immunoassay
ELISA: Enzyme-linked immunosorbent assay
FSH: Follicle stimulating hormone
GC-MS: Gas chromatography mass spectrometry
GSM: Genitourinary syndrome of menopause
HR+: Hormone receptor positive
LC-MS: Liquid chromatography mass spectrometry
LH: Lutenising hormone
RCT: Randomised controlled trial
RIA: Radioimmunoassay
SBHG: Sex hormone-binding globulin
VE: Vaginal oestrogen
